# Cardiovascular Effects, Phytochemistry, Drug Interactions, and Safety Profile of *Foeniculum vulgare* Mill. (Fennel): A Comprehensive Review

**DOI:** 10.3390/ph18111761

**Published:** 2025-11-19

**Authors:** Amal Zahi, Amama Rani, Nahida Aktary, Muntajin Rahman, Hassane Mekhfi, Abderrahim Ziyyat, Moon Nyeo Park, Abdelkhaleq Legssyer, Bonglee Kim

**Affiliations:** 1 Laboratory of Bioresources, Biotechnology, Ethnopharmacology and Health, Faculty of Sciences, Mohammed First University, Oujda 60000, Morocco; amal.zahi@ump.ac.ma (A.Z.); h.mekhfi@ump.ac.ma (H.M.); ziyyat@yahoo.fr (A.Z.); a.legssyer@ump.ac.ma (A.L.); 2 Department of Pathology, College of Korean Medicine, Kyung Hee University, Seoul 02447, Republic of Korea; amama.rani@khu.ac.kr (A.R.); nahidabph@gmail.com (N.A.); muntajinrahman899@gmail.com (M.R.); mnpark@khu.ac.kr (M.N.P.); 3 Korean Medicine-Based Drug Repositioning Cancer Research Center, College of Korean Medicine, Kyung Hee University, Seoul 02447, Republic of Korea

**Keywords:** cardiovascular health, *Foeniculum vulgare* Mill., trans-anethole, fenchone, flavonoids, phenolic acids, drug interactions, toxicity

## Abstract

**Background/Objectives:** Cardiovascular diseases remain the leading cause of mortality worldwide. According to the World Heart Federation, more than 500 million people were living with cardiovascular diseases in 2021. In this context, the use of medicinal plants has become increasingly widespread in populations as a preventive strategy against cardiovascular disorders. *Foeniculum vulgare* Mill., commonly known as fennel, is an aromatic and medicinal plant recognized for its beneficial properties in the treatment of various ailments, due to its richness in bioactive compounds. This review aims to summarize and analyze the cardiovascular activities of this plant, based on experimental evidence, and to provide an updated overview of its phytochemical composition and safety profile. **Methods:** A comprehensive literature search was conducted using databases including PubMed, Scopus, Web of Science, and Google Scholar, encompassing all publications available up to 2024. This search included research articles, reviews, mini-reviews, and clinical studies published in English. Exclusion criteria comprised publication types such as letters, conference abstracts, unpublished theses, and non-peer-reviewed reports. Studies were also excluded if they did not specifically address *Foeniculum vulgare* Mill. or its cardiovascular activities. All studies were screened according to predefined inclusion and exclusion criteria, and relevant data were systematically extracted and analyzed to synthesize current knowledge on the cardiovascular activities, mechanisms of action, phytochemical composition, safety, and potential drug interactions of *Foeniculum vulgare* Mill. **Results:** Numerous in vitro and in vivo studies have demonstrated that *Foeniculum vulgare* Mill. exhibits a wide range of activities beneficial for cardiovascular health. These include antihypertensive, cardioprotective, vasorelaxant, anti-inflammatory, antioxidant, diuretic, hypotensive, hypolipidemic, antiplatelet, and anticoagulant effects. Such pharmacological actions are largely attributed to its rich phytochemical composition, particularly its volatile oils (e.g., trans-anethole, fenchone), flavonoids (e.g., quercetin, kaempferol), and phenolic acids (e.g., *p*-coumaric acid, ferulic acid). Most studies report no significant signs of toxicity. **Conclusions:** *Foeniculum vulgare* Mill. emerges as a promising medicinal plant for the prevention and management of cardiovascular diseases, owing to its multifaceted beneficial effects and its favorable safety profile. However, potential interactions with cardiovascular drugs and the current limitations of existing studies highlight the need for further clinical research to fully establish its therapeutic potential.

## 1. Introduction

Cardiovascular disorders (CVDs) are among the leading causes of death and significantly impair the quality of life worldwide. In 2019, cardiovascular diseases were responsible for 17.9 million deaths worldwide, accounting for 32% of global deaths, according to the World Health Organization [1]. Among these deaths, 85% were attributed to heart attacks and strokes. Data from the World Heart Federation in 2021 indicated that over 500 million people were living with cardiovascular diseases [2]. Cardiomyopathy (CMD), coronary artery disease (CAD) and heart failure (HFD) are characterized by unique pathophysiological changes that drive their specific clinical outcomes [3]. Guidelines for prevention of CVD list hypertension, abnormal lipid levels, smoking and diabetes mellitus as the primary modifiable risk factors [4]. Lifestyle modifications such as limiting tobacco and alcohol use, following a balanced diet low in fat and sugar, and staying physically active to prevent obesity can reduce incidence. However, the risk of CVDs increases with age and the presence of contributing factors such as oxidative stress, chronic inflammation, and a family history of heart attacks [5,6]. Medicinal plants contain diverse bioactive compounds such as flavonoids, alkaloids and polyphenols which exhibit significant therapeutic and health-promoting properties [7]. Investigating traditionally used plants is crucial to ensure treatment effectiveness, aiming to use them for prevention and alongside conventional chemical drugs [8].

*Foeniculum vulgare* Mill. (FV), commonly known as fennel, is a medicinal and aromatic plant from the Apiaceae (Umbelliferae) family. It has been used since ancient times, valued both as a culinary spice and for its wide range of therapeutic properties [9]. Numerous studies have demonstrated the efficacy of fennel in managing various health conditions, including inflammation, hyperlipidemia, hypertension, and others cardiovascular disorders [10,11,12,13]. The present review aims to compile and critically analyze the diverse biological activities of FV, with particular emphasis on its cardiovascular activities, as well as its botanical characteristics, phytochemical composition, potential drug interactions, and safety profile. By integrating current scientific findings, this review provides a comprehensive overview of the cardiovascular properties and underlying mechanisms of FV, thereby supporting its traditional use in the prevention and management of cardiovascular diseases. However, it is important to clinically validate its cardiovascular effects in order to establish its therapeutic potential.

## 2. Methodology of Research

To collect relevant data, a comprehensive literature search was conducted across multiple electronic databases, including PubMed, Scopus, Web of Science, and Google Scholar, covering all available publications up to 2024. The search strategy employed keywords such as *Foeniculum vulgare* Mill., fennel, botanical characteristics, cardiovascular pharmacology, vasorelaxation, antihypertensive, cardioprotective, diuretic, antioxidant, anti-inflammatory, antiaggregant, anticoagulant, hypolipidemic, phytochemical composition, potential drug interactions, and toxicity. The inclusion criteria covered original research articles, book chapters, reviews, mini-reviews, and clinical studies published in English that specifically focused on *Foeniculum vulgare* Mill. The exclusion criteria comprised letters, conference abstracts, unpublished theses, and non-peer-reviewed reports. The bibliographic data from the selected studies were extracted, analyzed, and synthesized to provide a comprehensive overview of the cardiovascular activities, underlying mechanisms, key bioactive compounds, drugs interactions, and safety profile of fennel. The chemical structures of the compounds presented in this review were drawn using ChemDraw software, version 8.

## 3. Botanical Description of *Foeniculum vulgare* Mill.

**Figure 1:**
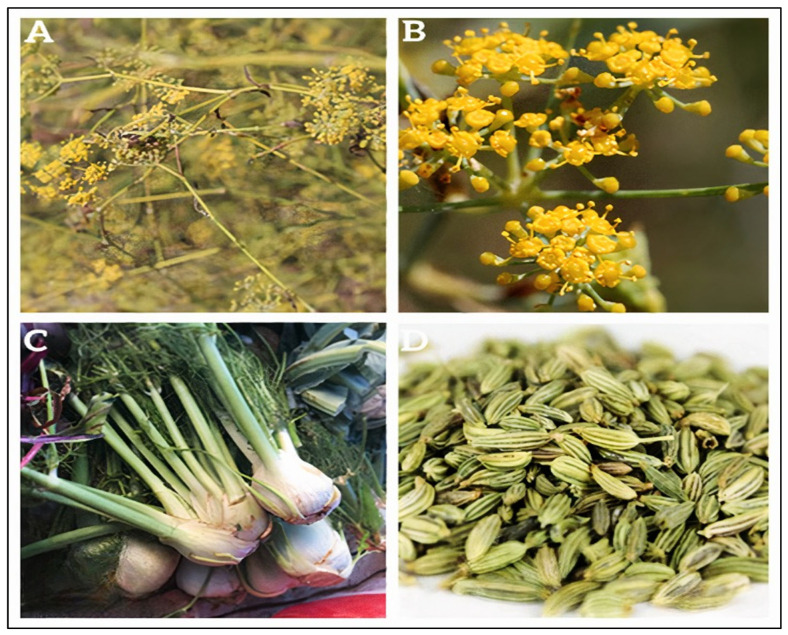
Different parts of *Foeniculum vulgare* Mill., (**A**) Branched stem bearing umbels; (**B**) Umbel inflorescence; (**C**) bulbs; (**D**) Seeds. (Photos were taken in northeastern Morocco).

*Foeniculum vulgare* Mill. is one of the oldest known medicinal and aromatic plants, traditionally valued for its wide range of therapeutic properties. It comprises two main botanical varieties: *Foeniculum vulgare* var. *dulce* (sweet fennel) and *Foeniculum vulgare* var. *vulgare* (bitter fennel), both widely used in traditional and modern medicine. Historical records even cite an ancient belief attributed to Adam, suggesting that consuming fennel with sugar daily during spring could protect against all diseases, underscoring its long-standing importance in traditional health practices [14]. Fennel is a hardy, perennial, and aromatic herb that can reach up to 2.5 m in height. It has erect, hollow stems, and its leaves are finely dissected and feathery, reaching up to 40 cm in length, with thread-like segments approximately 0.5 mm wide. The plant produces bright yellow flowers arranged in large compound umbels at the tips of the stems [15]. The fruit, often referred to as a seed, is actually a dry schizocarp composed of two mericarps (also called achenes). These are elongated, oval, and ribbed, initially bluish in color and turning brownish-grey upon maturation. The typical size of the fennel fruit is 4–10 mm long and about 3 mm wide, though it may vary depending on growth stage and environmental conditions [16]. Fennel seeds are aromatic and flavorful, widely used as a spice and in herbal remedies. The plant is green to white in color, with bulbous leaf bases that may resemble a swollen stem, especially in cultivated varieties (Figure 1) [17]. It grows wild across much of temperate Europe but is generally regarded as native to the Mediterranean region. Fennel is known for producing a variety of valuable secondary metabolites, used in pharmaceuticals, agrochemicals, fragrances, dyes, biopesticides, and as food additives [18].

## 4. Phytochemistry of *Foeniculum vulgare* Mill.

Phytochemical investigations of FV have identified a wide array of bioactive constituents, including volatile oils, phenolic compounds, flavonoids, coumarins, alkaloids, terpenoids, glycosides, tannins, and other secondary metabolites [19]. The concentration and composition of these metabolites vary substantially depending on the plant’s morphological characteristics, growth stage, and geographical origin. All parts of the plant like roots, leaves, fruits, and particularly seeds are utilized for diverse therapeutic and culinary purposes [20]. The whole FV plant has been reported to contain approximately 6.3% moisture, 9.5% protein, 10% fatty acids, 13.4% minerals, 18.5% fiber, and 42.3% carbohydrates. Its mineral and vitamin profile includes calcium, potassium, sodium, iron, and phosphorus, as well as thiamine, riboflavin, niacin, and vitamin C. The plant’s calorific value is estimated at 370 kcal per 100 g [21].

### 4.1. Volatile and Non-Volatile Compounds

**Figure 2:**
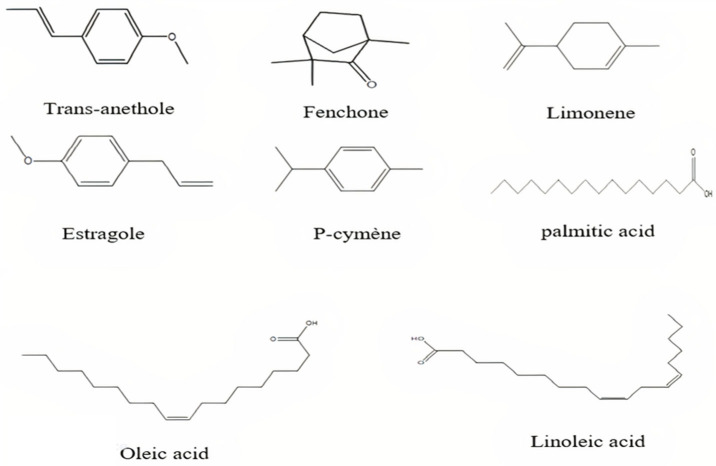
Chemical structures of the main volatile and non-volatile compounds of *Foeniculum vulgare* Mill. (ChemDraw 8.0).

FV is renowned for its characteristic aroma, primarily attributed to its essential oil, which is widely used as a flavoring agent in culinary and pharmaceutical preparations. The essential oil is dominated by trans-anethole (50–70%), a major phenylpropanoid compound, followed by notable amounts of estragole (10–40%), fenchone (3–10%), and limonene (2–11%). Several minor constituents also contribute to its complex chemical profile [20,22,23,24,25,26,27,28,29,30,31,32]. The relative proportions of these volatile compounds vary considerably depending on factors such as geographical origin, cultivation conditions, developmental stage, and fruit ripeness. For instance, GC-MS analyses have revealed marked regional variations: estragole predominates in Egyptian fennel seeds (51.04%), whereas trans-anethole is the major component in Chinese (54.26%) and Iraqi (43.13%) samples [27,28]. Furthermore, it has been found that fennel fruits contain a complex mixture of volatile constituents representing more than 98% of the total essential oil content. Among these, estragole levels may range from 34% to 89%, depending on environmental factors [32]. The fatty acid profile of fennel fruit oil comprises approximately 4% palmitic acid, 22% oleic acid, 14% linoleic acid, and 6% petroselinic acid. These compounds are distributed throughout the seeds, roots, stems, flowers, and fruits, with varying concentrations across the different plant tissues [20]. The chemical structures of the main volatile and non-volatile compounds are illustrated in Figure 2.

### 4.2. Phenolic Compounds

FV also contains a variety of phenolic compounds, such as flavonoids, phenolic acids (hydroxycinnamic and hydroxybenzoic acids), coumarin, and tannin [14]. These compounds are believed to contribute to the prevention of diseases linked to oxidative stress, such as cardiovascular diseases, cancer, and inflammation [33].

#### 4.2.1. Flavonoids

**Figure 3:**
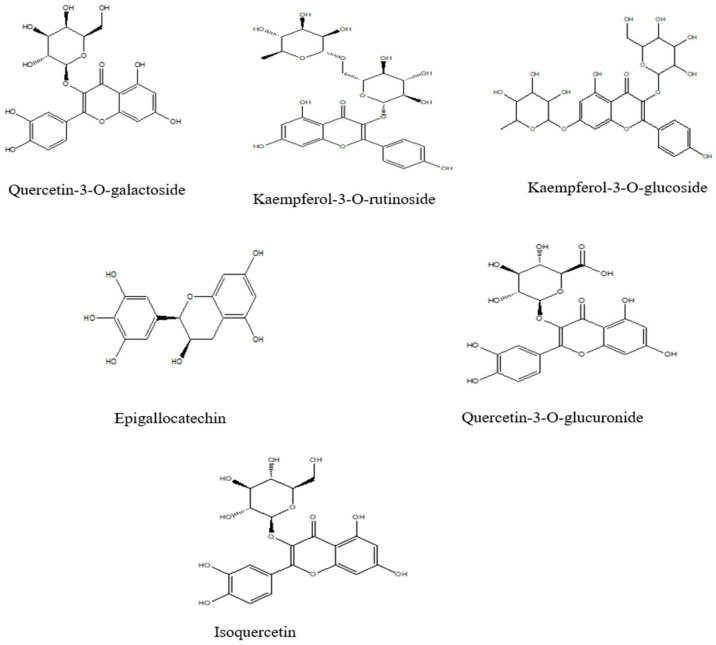
Chemical structures of the main flavonoids of *Foeniculum vulgare* Mill. (ChemDraw 8.0).

Flavonoids such as eriodictyol-7-rutinoside and quercetin-3-rutinoside have been isolated from FV. In addition, its aqueous extract contains quercetin-3-O-galactoside, kaempferol-3-O-rutinoside, and kaempferol-3-O-glucoside. Further studies have identified quercetin-3-O-glucuronide, kaempferol-3-O-glucuronide, isoquercetin, and isorhamnetin-3-O-glucoside as additional flavonoid constituents of FV [34]. HPLC-DAD analysis of the butanolic fraction of fennel seeds also revealed the presence of epigallocatechin, vanillin (a phenolic aldehyde), rutin (a flavonoid glycoside), and naringin [12]. The chemical structures of the major flavonoids are illustrated in Figure 3.

#### 4.2.2. Phenolic Acids

**Figure 4:**
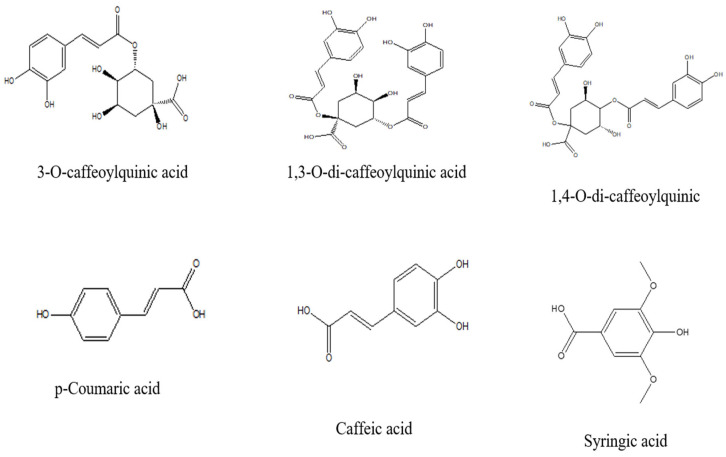
Chemical structures of the main phenolic acids of *Foeniculum vulgare* Mill. (ChemDraw 8.0).

FV has been reported to contain a wide range of phenolic acids, including 3-O-caffeoylquinic acid, 4-O-caffeoylquinic acid, 5-O-caffeoylquinic acid, 1,3-O-dicaffeoylquinic acid, 1,4-O-dicaffeoylquinic acid, and 1,5-O-dicaffeoylquinic acid [35]. Moreover, HPLC-DAD analysis of the butanolic fraction of fennel seeds revealed the presence of hydroxybenzoic acids, such as syringic acid, and hydroxycinnamic acids, including chlorogenic acid, caffeic acid, *p*-coumaric acid, and ferulic acid [12]. The chemical structures of the principal phenolic acids are illustrated in Figure 4.

#### 4.2.3. Coumarins

**Figure 5:**
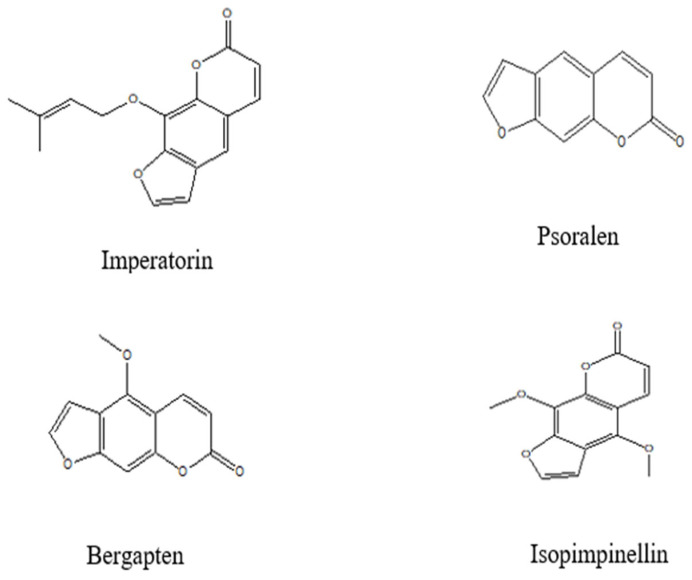
Chemical structures of the main coumarin of *Foeniculum vulgare* Mill. (ChemDraw 8.0).

Using TLC analysis, several coumarins such as imperatorin, psoralen, bergapten, xanthotoxin, and isopimpinellin were isolated from the methylene chloride extract of FV fruits [36]. Similarly, four coumarin compounds, namely scopoletin, 8-methoxypsoralen, bergapten, and imperatorin, were identified in the methanolic extract of fennel fruits [37]. The chemical structures of the major coumarins are presented in Figure 5 below.

## 5. Cardiovascular Activities of *Foeniculum vulgare* Mill.

**Figure 6:**
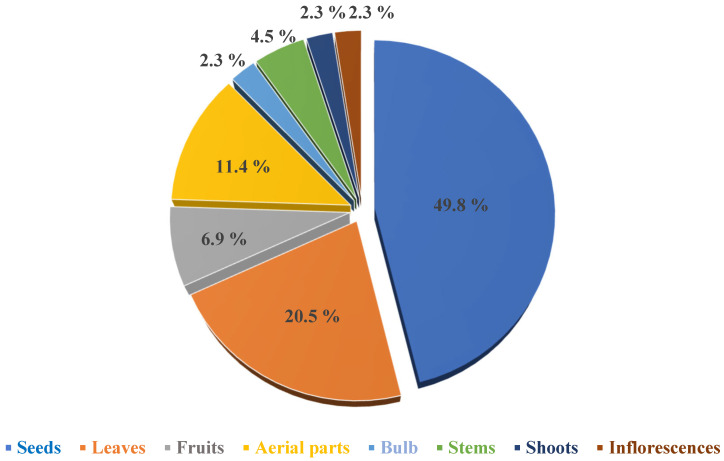
Illustration showing the percentage of *Foeniculum vulgare* Mill. parts used in experimental studies investigating its cardiovascular-related effects.

Table 1 summarizes studies investigating the cardiovascular activities of FV, including the plant parts used, tested bioactive compounds, extract types and administered doses, experimental models employed, types of tests conducted, and the cardiovascular outcomes reported in each study. Collectively, these experimental findings indicate that FV exhibits significant potential in cardiovascular protection, attributed to its hypotensive, antihypertensive, vasorelaxant, hypolipidemic, antithrombotic, anticoagulant, antioxidant, diuretic, cardioprotective, and anti-inflammatory activities. Among the various plant parts evaluated, the seeds are the most frequently used in experimental studies (43%), followed by the leaves (20.5%), aerial parts (11.4%), fruits (6.9%), stems (4.5%), bulbs (2.3%), shoots (2.3%), and inflorescences (2.3%), as illustrated in Figure 6.

### 5.1. Hypotensive Activity

Several studies have reported the hypotensive potential of FV extracts using different experimental models. The aqueous seed extract of FV (FVE) significantly reduced intraocular pressure (IOP) in normotensive rabbits, showing effects comparable to timolol [38]. This activity has been attributed to its anticholinesterase effect, which increases acetylcholine availability, enhances aqueous humor outflow, and consequently decreases IOP [77]. Similarly, the decocted aqueous extract of FV leaves (12 mg/kg/day) reduced arterial blood pressure in normotensive rats without affecting pulse or respiratory rate. This hypotensive effect appears to involve histaminergic pathways, whereas cholinergic, serotonergic, ganglionic, and adrenergic mechanisms were not implicated [39]. Comparable hypotensive effects were also observed for FV essential oil constituents such as estragole and trans-anethole, which induced dose-dependent decreases in mean arterial pressure and heart rate in normotensive rats [78]. Likewise, monoterpenes including (+)-α-pinene, (–)-β-pinene, (±)-citronellol, and (±)-linalool produced hypotension accompanied by reflex tachycardia, suggesting peripheral vasodilation and baroreflex activation [79]. Taken together, these findings suggest that FV exerts its hypotensive effect through multiple mechanisms, likely involving both central and peripheral pathways, partly mediated by its major volatile and non-volatile constituents.

### 5.2. Antihypertensive Activity

Several studies have demonstrated that FV and its bioactive compounds exert antihypertensive effects mainly through inhibition of the renin-angiotensin system, particularly the angiotensin-converting enzyme (ACE) and angiotensin II receptor pathways. In vitro investigations revealed that both the essential oil and its major constituent anethole significantly inhibited ACE activity, with IC_50_ values of 40.7 ± 3.5 µg/mL and 52 ± 5.8 µg/mL, respectively [44]. Consistently, methanolic extracts of FV leaves inhibited ACE activity by about 50% [45]. Similar effects were observed for phenolic acids such as gallic, caffeic, and coumaric acids, which exhibited substantial ACE inhibitory potential, with caffeic acid showing the strongest effect (IC_50_ = 157.3 ± 16.1 µM) [80]. Other polyphenols, including kaempferol and quercetin, have also been shown to inhibit ACE activity and downregulate the expression of the angiotensin II type 1 receptor (AT1R), both in vitro and in vivo [81,82,83]. Molecular docking analyses confirmed these findings, showing a strong binding affinity of quercetin to the active site of human ACE (ΔG = −8.1 kcal/mol) [84]. Likewise, naringin was reported to inhibit ACE activity and suppress angiotensin II-induced gene expression [85]. In vivo, the aqueous extract of FV fruits (190 mg/kg/day) significantly lowered systolic blood pressure in spontaneously hypertensive rats but not in normotensive controls, suggesting a selective effect under pathological conditions [40]. This extract also promoted diuresis by increasing water, sodium, and potassium excretion. Similar blood pressure reductions were observed with nanoemulsions rich in FV phenolic compounds, which significantly decreased mean, systolic, and diastolic blood pressure while normalizing heart rate in salt-induced hypertensive rats [47]. Additionally, treatment with trans-anethole reduced AT1R expression, further supporting the role of FV constituents in modulating the renin-angiotensin system [86]. These results suggest that FV exerts its antihypertensive effect primarily through inhibition of ACE activity, downregulation of AT1R expression, and enhancement of renal excretory function by its bioactive constituents.

### 5.3. Diuretic Activity

Several studies have demonstrated that FV exhibits notable diuretic activity through multiple mechanisms involving increased urine output and modulation of electrolyte excretion. The ethanolic extract of FV fruits (500 mg/kg) significantly enhanced diuresis in rats at both 5 and 24 h post-administration, producing an effect comparable to urea (960 mg/kg) and nearly doubling urine output compared to controls, without altering sodium or potassium excretion [49]. Consistently, both aqueous and methanolic extracts of FV leaves (200–400 mg/kg) induced a dose-dependent increase in urine volume and significantly enhanced natriuresis, kaliuresis, and chloriuresis [50]. At the phytochemical level, several FV-derived compounds also contribute to its diuretic action. Phenolic acids such as chlorogenic and caffeic acids increased urine volume and sodium excretion, while rosmarinic acid displayed a potassium-sparing effect [87]. Similarly, the fenchone (400 mg/kg) enhanced urine output and electrolyte (Na^+^, K^+^, Ca^2+^) excretion in a dose-dependent manner [88]. Flavonoids such as isoquercitrin and (−)-epicatechin further promoted diuresis and saluresis (Na^+^, K^+^, Cl^−^) without disturbing plasma electrolyte balance or urinary pH [89,90]. These findings indicate that FV promotes diuresis through synergistic actions of its phenolic, flavonoid, and monoterpene constituents, acting via osmotic and ion-transport modulation mechanisms.

### 5.4. Vasorelaxant Activity

Several studies have demonstrated that FV exerts significant vasorelaxant effects through multiple endothelium-dependent and -independent mechanisms involving nitric oxide (NO), calcium modulation, and potassium channel activation. In vivo, treatment with the aqueous extract of FV fruits (190 mg/kg/day for 4 days) reduced noradrenaline-induced aortic contraction by 19 ± 6.2% in SHR, but not in normotensive rats. The effect was abolished by Nω-Nitro-L-arginine methyl ester (L-NAME), indicating NO-mediated relaxation [40]. Similarly, aqueous and methylene chloride fractions of FV extracts induced concentration-dependent vasodilation in mesenteric arteries and isolated rat aorta, respectively, mediated via the NO/cGMP pathway and muscarinic receptor activation [12,43]. The butanolic fraction, rich in phenolic acids (coumaric, syringic, ferulic) and the flavonoid naringin, exhibited the strongest vasorelaxant activity in mesenteric arteries. Increased nitrite and cGMP levels following FV seed extract treatment further support enhanced endothelial NO production [42]. Essential oil and its major compound trans-anethole (1–300 µg/mL) also produced complete, concentration-dependent relaxation of phenylephrine- and KCl-induced contractions in isolated rat aorta, independent of the endothelium [41,46]. These effects may involve inhibition of store-operated and voltage-dependent Ca^2+^ entry, suppression of IP_3_-dependent Ca^2+^ release, and blockade of non-selective cation channels and phospholipase C activation [46]. In addition, FV phenolic and flavonoid constituent such as caffeic, ferulic, and coumaric acids, quercetin, kaempferol, and naringenin have demonstrated potent vasodilatory properties. These compounds act via activation of the NO/sGC/cGMP pathway, modulation of Ca^2+^ influx, and opening of potassium channels, with both endothelium-dependent and independent effects [91,92,93,94]. Overall, FV induces vascular relaxation via complementary mechanisms, including NO-mediated pathways, calcium channel blockade, and potassium channel activation, primarily mediated by its vasoactive compounds.

### 5.5. Cardioprotective Activity

Treatment with hydroalcoholic extracts of FV fruits (200–400 mg/kg, 28 days) significantly protected the myocardium against isoproterenol-induced injury in rats, as evidenced by normalized cardiac marker enzymes, improved lipid and glucose profiles, elevated glutathione levels, and histological signs of myocardial regeneration [13]. Similarly, preincubation of H9C2 cardiomyocytes with ethanolic extracts or anethole markedly reduced oxidative stress, DNA damage, and mitochondrial dysfunction following hypoxia/reoxygenation injury [48]. Several bioactive constituents of FV also demonstrated cardioprotective potential. Caffeic acid phenethyl ester derivatives (3–15 mg/kg) attenuated ischemia/reperfusion injury in rabbits by suppressing MAPK phosphorylation and proinflammatory cytokine expression (IL-1β, TNF-α) [95,96,97,98,99]. Syringic acid (50 mg/kg) protected against isoproterenol-induced cardiotoxicity over 30 days in rats [99], while S-limonene (1 mg/kg) prevented ECG abnormalities, infarct size, and collagen deposition, likely via the inhibition of calcium overload and oxidative stress, and the restoration of antioxidant enzyme activity [100]. In addition, flavonoids such as quercetin and catechin enhanced cardiac recovery after ischemia/reperfusion by reducing inflammatory cytokines (IL-1β, TNF-α, IL-6), improving mitochondrial function, and limiting apoptosis through signaling pathways involving CREB/lncRNA MIAT/Akt/GSK-3β [101,102]. These findings indicate that FV confers cardioprotective effects through multiple complementary pathways, involving antioxidant, anti-inflammatory, and antiapoptotic mechanisms, along with the regulation of calcium homeostasis and preservation of mitochondrial function

### 5.6. Anticoagulant and Antithrombotic Activities

Several studies have demonstrated that FV and its main constituents exert potent antiplatelet and anticoagulant activities through multiple mechanisms. The essential oil and its major components, particularly (+)-fenchone, estragole, and anethole, significantly inhibited platelet aggregation induced by collagen, arachidonic acid (AA), and ADP in vitro, with effects comparable to those of acetylsalicylic acid (ASA). These compounds also reduced thromboxane A_2_ mediated aggregation and prevented thrombin-induced clot retraction, leading to marked antithrombotic protection in vivo, where anethole and the essential oil (30 mg/kg/day) prevented 83% and 70% of paralysis events in a mouse thromboembolism model, respectively [41,64]. Polyphenolic compounds such as chlorogenic, *p*-coumaric, and syringic acids further contributed to these effects. Chlorogenic acid inhibited fibrin clot formation in a dose-dependent manner, prolonged prothrombin time (PT), and enhanced fibrinolysis [103]. Similarly, *p*-coumaric acid suppressed ADP- and AA-induced platelet aggregation in rabbits and human blood via decreased thromboxane B_2_ and prostaglandin E_2_ production [104]. Syringic acid also inhibited fibrin clot formation and reduced factor Xa activity, suggesting direct inhibition of procoagulant enzymes [105]. In addition, flavonoids such as quercetin and catechin displayed strong synergistic antiplatelet effects. Quercetin completely inhibited AA-induced aggregation and reduced thrombin-mediated platelet activation by suppressing Ca^2+^ mobilization and serotonin release. Together with catechin, it downregulated platelet GPIIb/IIIa expression through nitric oxide-dependent pathways [106,107]. In summary, these results suggest that FV exerts anticoagulant and antithrombotic effects through combined inhibition of platelet activation and aggregation, modulation of the arachidonic acid and nitric oxide pathways, and direct suppression of coagulation enzymes.

### 5.7. Anti-Inflammatory Activity

Numerous studies have shown that FV exhibits potent anti-inflammatory activity through the inhibition of key inflammatory mediators, signaling pathways, and enzymes. At the cellular and molecular level, the methylene chloride and ethanolic extracts of FV fruits significantly suppressed LPS-induced nitric oxide production and downregulated iNOS and COX-2 expression in RAW 264.7 macrophages. These extracts also reduced the production of pro-inflammatory cytokines such as TNF-α, IL-1β, and IL-6, partly through inhibition of JNK1 and ERK1/2 phosphorylation [43,54]. Similarly, aqueous and hydromethanolic extracts decreased NF-κB activation, IκB-α phosphorylation, and NO release in microglial BV-2 cells, confirming their regulatory effects on NF-κB signaling and cytokine production [61,62]. In vivo studies further demonstrated that FV extracts alleviate acute and chronic inflammation. Methanolic and essential oil extracts markedly inhibited carrageenan- and arachidonic acid-induced paw and ear edema in rodents (up to 70% inhibition), while α-pinene and anethole exhibited comparable effects to indomethacin [10,56,57]. Fennel essential oil (200–400 mg/kg) also protected against acetic acid-induced colitis by reducing MPO activity, TNF-α expression, and NF-κB p65 phosphorylation [58]. Additionally, aqueous seed extract attenuated intestinal inflammation in necrotizing enterocolitis, lowering caspase activation and inflammatory cytokine levels [60]. Selenium nanoparticles synthesized from fennel seeds significantly reduced arthritis severity and joint damage in arthritic mice [55]. Polyphenolic compounds such as quercetin, ferulic acid, chlorogenic acid derivatives, and *p*-coumaric acid contribute to the anti-inflammatory activity. These compounds inhibit COX-2 selectively, suppress NF-κB and NLRP3 inflammasome activation, and downregulate TNF-α, IL-6, and IL-1β expression [59,108,109,110]. Notably, quercetin glucoside, dicaffeoylquinic acid, and isorhamnetin glucuronide showed strong selective COX-2 inhibition with IC_50_ values between 9 and 16 µM [59]. Anethole and estragole also displayed anti-inflammatory potential in carrageenan models and modulated Th17/Treg immune balance [111,112]. These findings indicate that FV exerts its anti-inflammatory activity via several complementary mechanisms, including inhibition of NF-κB and MAPK signaling, suppression of iNOS and COX-2 expression, downregulation of pro-inflammatory cytokines, and modulation of immune cell responses.

### 5.8. Hypolipidemic Activity

Several experimental studies have demonstrated the hypolipidemic potential of FV through modulation of lipid metabolism, antioxidant protection, and regulation of cholesterol homeostasis. In obese male rats, oral administration of FV seed powder (300 mg/kg) for six weeks significantly reduced body weight, serum cholesterol, triglycerides, LDL, albumin, and total protein, while increasing HDL levels. Treatment also lowered liver enzyme activities (ALT, AST, ALP) and oxidative stress markers such as MDA and MPO, indicating hepatoprotective and antioxidant effects [65]. Similarly, intraperitoneal administration of aqueous seed extracts (50–200 mg/kg) for 14 days in BALB/c mice fed a cholesterol-enriched diet led to a significant decrease in total cholesterol levels, triglycerides, and LDL, and a dose-dependent increase in HDL levels. The extract also upregulated leptin receptor expression, suggesting improved lipid and energy metabolism regulation [11]. In rats fed a high-cholesterol diet, hydroalcoholic seed extract (150 mg/kg, i.p., 3 weeks) significantly reduced triglycerides, total cholesterol, LDL, LDH, ALT, and ALP, while increasing HDL levels, confirming its lipid-lowering and hepatoprotective activity [66]. At the molecular level, bioactive compounds from FV, including quercetin, ferulic acid, *p*-coumaric acid, and trans-anethole, contribute to its hypolipidemic effect through antioxidant and regulatory mechanisms. Quercetin has been shown to reduce erythrocyte cholesterol by up to 75% in hypercholesterolemic subjects and to promote LDL uptake in HepG2 cells [113,114]. Phenolic acids such as ferulic and *p*-coumaric acids inhibit lipid peroxidation and improve oxidative status, while trans-anethole decreases ROS generation and LDL oxidation, resulting in higher HDL and lower VLDL levels [113,115]. Limonene supplementation reduced HMG-CoA reductase activity and prevented the conversion of lb-LDL to sd-LDL, thereby decreasing atherogenic risk [114]. In liver cell models, quercetin, catechin, hesperidin, and isorhamnetin significantly downregulated the expression of SREBP-2 and LDLR, key regulators of cholesterol synthesis and clearance [115]. Overall, the evidence indicates that FV exerts hypolipidemic effects via a combination of antioxidant, hepatoprotective, and metabolic regulatory mechanisms, involving inhibition of HMG-CoA reductase, attenuation of LDL oxidation, and modulation of lipid-related gene expression.

### 5.9. Antioxidant Activity

**Figure 7:**
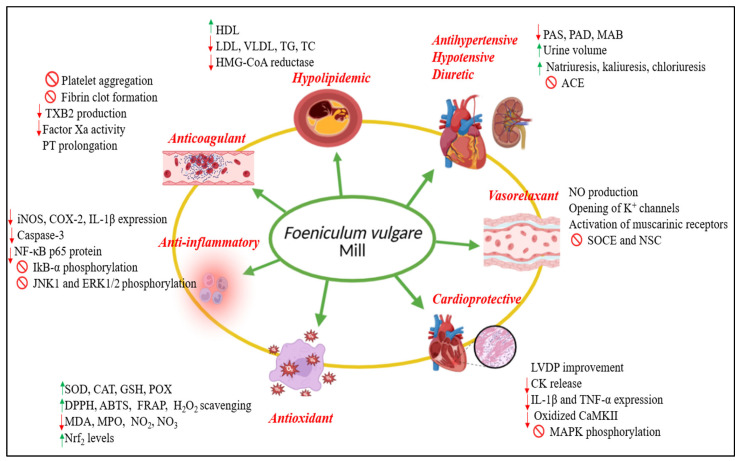
Comprehensive summary of the mechanisms underlying the cardiovascular activities of *Foeniculum vulgare* Mill. ⊘ Inhibition; 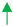 Increase; 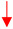 Decrease.

Several studies have demonstrated the strong antioxidant potential of FV in both in vivo and in vitro models. Administration of the methanolic fruit extract (200 mg/kg/day) for three weeks significantly increased SOD and catalase activities compared to the control group [10]. Similarly, the methanolic seed extract showed strong antioxidant activity, achieving nearly complete inhibition of DPPH absorption, and provided cytoprotection against γ-irradiation by restoring MDA, catalase, and glutathione levels [67]. In vitro assays confirmed the antioxidant efficacy of various FV extracts (aqueous, ethanol, acetone, and methanol), with the distilled water extract exhibiting the highest lipid peroxidation inhibition in the FTC and β-carotene bleaching assays, while the acetone extract showed the greatest ABTS radical scavenging capacity [68]. Essential oils obtained from aerial parts also displayed potent antioxidant activity, surpassing that of α-tocopherol except at the lowest concentration tested [69]. Likewise, essential oil, diethyl ether, and ethyl acetate leaf extracts demonstrated significant DPPH radical scavenging effects, with IC_50_ values of 900, 6.2, and 1.5 µg/mL, respectively [70], while the weak scavenging activity of fennel leaf oil was attributed to its anethole content [71]. Coumarins isolated from fennel fruits, like scopoletin, 8-methoxypsoralen, bergapten, and imperatorin exhibited strong DPPH and ABTS radical scavenging activities (50%) [37]. Methanolic extracts of fennel leaves, stems, shoots, and inflorescences displayed concentration-dependent increases in DPPH scavenging, reducing power, and β-carotene bleaching inhibition, with over 90% inhibition at higher doses [72]. Essential oils from aerial parts and fruits also showed high DPPH scavenging (>85%) and 5-lipoxygenase inhibition, though limited hydroxyl radical scavenging capacity [73]. Moreover, fennel leaf essential oil (EOF) reduced ABTS and H_2_O_2_ radicals by 50% and decreased ROS levels in leukocytes, accompanied by enhanced CAT, SOD, and GPx activities in a dose-dependent manner [74]. The hydromethanolic extract of fennel seeds increased hydroxyl radical scavenging activity up to 82.6% inhibition at 100 µg/mL [75]. Fennel fruit oil showed strong total antioxidant capacity (7.26 ± 0.34 mg GAE/g) and reducing power (FRAP EC_50_ = 63.44 ± 2.29 mg/mL) while reducing MDA and enhancing SOD and CAT activities in bleomycin-induced pulmonary fibrosis [76]. The aqueous and butanolic fractions of FV seeds also demonstrated potent, concentration-dependent antioxidant activities in DPPH and β-carotene assays, with butanolic fraction showing higher efficacy [12]. Other studies highlighted the contribution of individual compounds. α-Pinene and trans-anethole increased SOD, POX, CAT, and GST activities in insect models [116]. In colitis models, (–)-fenchone (150 mg/kg) elevated GSH and SOD while reducing MDA and MPO levels [117]. Flavonoids such as quercetin and kaempferol enhanced Nrf2 and antioxidant gene expression (SOD1, GPX3) and restored glutathione under oxidative stress [118,119]. Likewise, epicatechin and rutin exhibited potent radical scavenging abilities, while *p*-coumaric, chlorogenic, ferulic, and syringic acids provided additional antioxidant protection through ROS scavenging, metal chelation, and upregulation of endogenous defenses [120,121,122,123,124]. These findings suggest that the antioxidant potential of FV may result from the combined actions of its phenolic, flavonoid, and terpenoid constituents, which contribute to radical scavenging, metal chelation, and reinforcement of antioxidant defense systems. Figure 7 bellow presents the cardiovascular and biological activities of FV, highlighting the principal underlying mechanisms involved.

FV exerts its cardiovascular activities through multiple and complementary pathways, as summarized in Table 2. The table also highlights the bioactive compounds associated with these mechanisms.

## 6. Safety Profile of *Foeniculum vulgare* Mill.

Several studies have confirmed the non-toxic and safe nature of FV across different experimental models. In acute toxicity tests, ethanolic extracts administered to mice at doses up to 3 g/kg showed no mortality or major behavioral or physiological alterations, even at the highest concentration [125]. Only slight and transient reductions in locomotor activity and mild piloerection were observed [49]. Other solvent extracts (n-hexane, methylene chloride, ethyl acetate, methanol) were also found to be non-toxic up to 5.5 g/kg in mice [36], and repeated oral administration up to 2 g/kg for 10 days caused no neurological or systemic toxicity [126]. Similarly, the butanolic fraction (2 g/kg) produced no adverse effects or mortality over a 14-day follow-up [12]. The lethal dose 50 of anethole in rats is estimated at 2090 mg/kg, and that of fennel essential oil at 1326 mg/kg. Subchronic exposure to one-third of this dose (695 mg/kg) induced only mild hepatic alterations, while long-term dietary administration caused no liver damage, confirming its low hepatotoxic potential [127]. However, some caution is warranted regarding estragole, a minor constituent of fennel oil, which has shown dose-dependent genotoxic and carcinogenic potential in certain models [128,129,130,131]. Due to its estrogenic activity, fennel preparations used for dysmenorrhea have also raised questions about possible teratogenic risks, though studies report no teratogenic effects up to 9.3 mg/mL [132]. Interestingly, anethole exhibits anti-genotoxic properties against several mutagens [133]. Finally, a 90-day subchronic study in rats receiving daily doses up to 1.56 g/kg confirmed the absence of chronic toxicity, supporting the plant’s long-term safety [134]. According to the U.S. Food and Drug Administration (FDA), FV is classified as a Generally Recognized As Safe (GRAS) substance under Title 21 CFR §182.10, as listed in the “Substances Added to Food” database (formerly EAFUS). This recognition supports the safety of FV for food and medicinal applications [135].

## 7. Interactions Between *Foeniculum vulgare* Mill. and Cardiovascular Drugs

It has been demonstrated that FV inhibits cytochrome P450 3A4 (CYP3A4). Consequently, fennel may reduce the metabolism of several cardiovascular drugs that are primarily metabolized by this enzyme, which could alter their bioavailability and therapeutic efficacy. Among these drugs are diuretics (e.g., eplerenone, spironolactone, indapamide), whose reduced metabolism may increase plasma concentrations, thereby enhancing the diuretic effect and potentially leading to electrolyte imbalances such as hypokalemia or hyponatremia [136,137]. For calcium channel blockers (e.g., amlodipine, diltiazem, felodipine, isradipine, nifedipine, nimodipine, nitrendipine, nisoldipine, verapamil), inhibition of CYP3A4 by fennel may cause elevated plasma levels, resulting in excessive vasodilation, hypotension, or bradycardia [136,138,139]. Similarly, β blockers (e.g., betaxolol, bisoprolol, carvedilol) may exhibit increased plasma concentrations due to decreased metabolic clearance, which could enhance their pharmacological effects and increase the risk of fatigue, bradycardia, or excessive hypotension [136,140,141].

## 8. Limitations and Future Perspectives

Despite the promising findings highlighting the cardiovascular potential of FV, most of the available data are derived from in vitro and in vivo studies. However, these experimental results remain largely unconfirmed by clinical investigations in humans. Moreover, the pharmacokinetic and pharmacodynamic profiles of the plant’s bioactive constituents have not been fully elucidated, limiting our understanding of their absorption, metabolism, and mechanism of action. Comprehensive clinical trials and detailed pharmacological evaluations are therefore essential to validate these preclinical results and ensure the safety and efficacy of FV in cardiovascular therapy.

## 9. Conclusions

Experimental studies demonstrate that FV exhibits a wide spectrum of pharmacological and biological activities beneficial for cardiovascular health. These include hypotensive, diuretic, antihypertensive, cardioprotective, vasorelaxant, anti-inflammatory, antioxidant, hypolipidemic, and antiaggregant effects, all contributing to the maintenance of vascular and cardiac function. These effects are mainly attributed to its rich phytochemical composition, particularly flavonoids, phenolic acids, and volatile constituents, which act synergistically through multiple molecular pathways. Toxicological investigations indicate that FV extracts are generally safe and well tolerated at experimental doses, although caution should be exercised regarding the toxicity of estragole under conditions of chronic or high-dose exposure. Furthermore, possible pharmacological interactions between FV and cardiovascular drugs deserve further investigation to ensure safe therapeutic use. Overall, this review provides an integrated overview of the cardiovascular activities, phytochemical composition, potential drug interactions, and safety profile of FV. These findings scientifically support its traditional use and highlight its potential as a promising natural source for cardiovascular protection.

## Figures and Tables

**Table 1:** Biological and pharmacological studies on the cardiovascular activities of *Foeniculum vulgare* Mill.

Fennel’s Parts	Extracts (Compound)/Dose Used	Experimental Model/Assays Type	Cardiovascular Activity	References
**Seeds**	Aqueous extract 0.3, 0.6 and 1.2% (*w*/*v*)	Normotensive and glaucoma acute in chronic models of rabbits (In vivo)	**Hypotensive effect** 17.49, 21.16 and 22.03% decrease in intraocular pressure at 0.3%, 0.6% and 1.2%, respectively.	[38]
**Leaves**	Aqueous extract (12 mg/kg).	Normotensive rats (In vivo)	**Hypotensive effect** Decrease in MBP in a dose-related manner without affecting the pulse or respiratory rate.	[39]
**Fruits**	Aqueous extract (190 mg/Kg/day)	Spontaneously hypertensive rats (SHR) and normotensive Wistar Kyoto rats (WKY) (In vivo)	**Antihypertensive and vasorelaxant effects** Reduction in the SBP of SHR but not of WKY. Increasing water, sodium and potassium excretion. NO-dependent vasorelaxation.	[40]
**Fruits and aerial parts**	Essential oil and anethole (1 to 300 µg/mL)	Rat isolated aorta (In vitro)	**Vasorelaxant effect** Fennel oil or anethole reduced phenylephrine or KCl induced contractions with IC_50_ ranging from 50 to 147 µg/mL. NO-independent vasorelaxation.	[41]
**Seeds**	Aqueous extract-derived nitrites (0.00069–1.38 µg/g)	Fertilized Chicken eggs (In vitro)	**Vasorelaxant effect** Significantly increases NO production and elevated the cGMP levels of endothelial cells.	[42]
**Fruits**	Methylene chloride fraction of crude methanolic extract (100–1000 µg/mL) 50,100, and 200 μg/mL (for inflammatory assay)	Isolated rat aortic rings (In vitro) RAW 264.7 macrophage cells (In vitro)	**Vasorelaxant effect** At 1000 μg/mL, a 69% vasodilatation was observed, which was endothelium-dependent. **Anti-inflammatory effect** Fennel extract at 100 and 200 µg/mL suppressed the expression of mRNA and proteins of both TNF-α and IL-1β in LPS-activated macrophages.	[43]
**Seeds**	Aqueous extract (0.01, 0.1, and 1) and sequential fractions of fennel (0.001–1 mg/mL)	Rat isolated mesenteric vascular beds (In vitro)	**Vasorelaxant effect** Fennel extract induced concentration endothelium dependent vasodilatory response with E_max_ = 81.73 ± 0.36%. The butanolic fraction showed the highest vasorelaxant effect by involvement of the NO/GMPc pathway, potassium channels, and muscarinic receptors.	[12]
**Fruits**	Essential oil and anethole (10–500 µg/mL)	ACE inhibition assay (In vitro)	**Antihypertensive effect** Both fennel oil and anethole showed significant ACE inhibition, with IC_50_ values of 40.7 ± 3.5 and 52 ± 5.8 µg/mL, respectively.	[44]
**Leaves**	Methanolic extract of phenolic compounds	ACE inhibition assay (In vitro)	**Antihypertensive effect** ACE inhibition activity of 50.8% was observed at a phenolic content of 17.04 mg/g.	[45]
**Seeds**	Oil and trans-anethole (0.01%)	Vascular endothelial cells (In vitro)	**Vasorelaxant effect** Fennel oil and trans-anethole inhibit SOCE in endothelial cells, which may involve the inhibition of NSC channels, IP_3_-dependent Ca^2+^ release, and PLC activation.	[46]
**Aerial part**	Nanoemulsion of phenolic compounds (1% and 2% *w*/*v*)	Salt-induced hypertensive rat model (In vivo)	**Antihypertensive effect** Significantly decreased MBP, SBP, DBP and normalized the heart rate in hypertensive rats at both doses.	[47]
**Fruits**	Hydroalcoholic extract (200; 250; and 400 mg/kg)	Isoproterenol (85 mg/kg, s.c) induced myocardial infarction in albino rats (In vivo)	**Cardioprotective effect** Significant reduction in serum levels of SGOT, SGPT, ALP, LDH, CPK, CRP, glucose, triglycerides, and the LDL/HDL ratio. Significant elevation in glutathione levels in both heart and liver. Myocardial cells regeneration.	[13]
**Seeds**	Ethanolic extract (0–100 µg/mL) Anethole (0–200 µM)	Hypoxia/reoxygenation injury in H9C2 heart myoblast cells (In vitro)	**Cardioprotective effect** Decrease in ROS generation, DNA double-strand break damage, nuclear condensation, and dissipation of mitochondrial membrane potential induced by hypoxia/reperfusion.	[48]
**Fruits**	Ethanolic extract (500 mg/kg)	Normotensive rats (In vivo)	**Diuretic effect** A highly significant diuretic effect was observed both at 5 h and 24 h after administration, without any change in sodium and/or potassium excretion.	[49]
**Leaves**	Aqueous and 80% methanol extracts (100, 200, 400 mg/kg)	Normotensive rats (In vivo)	**Diuretic effect** Both extracts increased urine volume after 24 h, natriuresis, kaliuresis, and chloriuresis at 200 and 400 mg/kg).	[50]
**Seeds**	Hydro-alcoholic extract (250–1000 mg/kg, oral)	Normal Wistar rats (In vivo)	**Immunomodulatory effect** The extract significantly increased RBC and WBC counts, particularly at a dose of 250 mg/mL, and CT at 500 mg/mL, compared to the control group.	[51]
**Fruits**	Methanolic extract (200 mg/kg, orally)	Carrageenan-induced paw edema, arachidonic acid-induced ear edema, formaldehyde-induced arthritis and type IV allergy (In vivo)	**Anti-inflammatory effect** Significant inhibition of paw edema (69%), and inhibition of ear edema (~70%). A Significant inhibitory effect on delayed-type hypersensitivity (immunosuppressive effect). **Antioxidant effect** Increase in SOD and CAT, and decreased levels of TBARS (lipid peroxidation).	[10]
**Leaves**	Ethanol extract (25.75, 51.50, 103, 206, and 412 μg/mL)	Membrane stabilization by induction of a hypotonic solution (In vitro)	**Anti-inflammatory effect** Significant inhibition of HRBC hemolysis, proportional to extract concentration, with an optimal concentration of 412 µg/mL.	[52]
**Seeds**	Ethanolic extract (CSE) Extraction with CO2 (SFE)	Carrageenan-induced paw edema (In vivo).	**Anti-inflammatory effect** Paw edema reduction (SFE: 30.43% CSE: 24.54%).	[53]
**Seeds**	Ethanolic extract (100 µg/mL)	RAW264.7 cells (In vitro)	**Anti-inflammatory effect** Inhibition of NO production: 78.70% ± 6.81% (IC_50_ = 47.91 µg/mL). Inhibition of TNF-α and IL-6 production: 42.21% ± 0.42% and 63.20% ± 1.04%, respectively.	[54]
**Seeds**	Selenium nanoparticles derived from fennel (5 and 10 mg/kg)	Arthritic Balb/c mice (In vivo).	**Anti-inflammatory effect** Reduction in paw volume at 5 mg/kg and at 10 mg/kg. Restored cellular morphology and no signs of erosion (5 and 10 mg/kg).	[55]
**Seeds**	Essential oil (0.050 and 0.200 mL/kg	Carrageenan induced rat paw edema (In vivo).	**Anti-inflammatory effect** Antiedema effect at 0.200 mL/kg (56.78% inhibition).	[56]
**Seeds**	Alpha-pinene (0.05, 0.10, 0.25 and 0.50 mL/kg)	Carrageenan induced rat paw edema (In vivo).	**Anti-inflammatory effect** Significant decrease in inflammation at 0.50 mL/kg (60.33%).	[57]
**Fruits**	Essential oil (100, 200, and 400 mg/kg)	Acute intestinal colitis induced by acetic acid (In vivo).	**Anti-inflammatory effect** Reduction of the ulcer index at 200 and 400 mg/kg. Reduction of the expression of TNF-α positive cells at 200 and 400 mg/kg. Reduction of the expression of p-NF-κB p65 protein at 400 mg/kg.	[58]
**Leaf, bulb, stem, and little stem**	Phenolic acids and glycosylated flavonoids (25, 50, 100, and 150 µM)	COX inhibitory fluorometric assay (In vitro).	**Anti-inflammatory effect** Kaempferol (IC_50_ = 228.38 ± 16.81 µM), isorhamnetin (IC_50_ = 94.72 ± 1.22 µM), and quercetin glucuronide (IC_50_ = 570.83 ± 40 µM) inhibited COX-1 enzymes more effectively than COX-2 enzymes.	[59]
**Seeds**	Aqueous extract (200 mg/kg/day)	Experimental necrotizing enterocolitis in rat (NEC) (In vivo).	**Anti-inflammatory effect** Significantly reduction in IL-6, TNF-α, and caspase-3 levels. Decrease in bowel injury.	[60]
**Fruits**	Aqueous extract (25, 50, and 100 µg/mL)	LPS-stimulated neuroinflammatory in BV-2 microglial cells (In vitro).	**Anti-inflammatory effect** Suppressed the expression of iNOS and COX-2 protein levels. NF-κB activation and IκB-α phosphorylation were inhibited in a dose-dependent manner. Fennel extract at 50 and 100 µg/mL significantly suppressed the increased expression of IL-6 and TNF-α.	[61]
**Seeds**	Hydromethanolic extract (50, 150, 200, and 250 µg/mL)	Protein denaturation, protease activity, membrane stabilization, and heat-induced hemolysis in RBC (In vitro).	**Anti-inflammatory effect** The maximum percentage of protein denaturation inhibition was 35.68 ± 0.40% at 200 µg/mL. The maximum inhibition of RBC hemolysis was 9.67 ± 0.30% at 200 µg/mL. Significantly higher protease inhibitory activity at 150, 200, and 250 µg/mL.	[62]
**Aerial parts**	Hexane, dichloromethane, butanol, and water fractions (0.01 to 200 µg/mL)	Proliferative lymphocytes by the BrdU incorporation assay (In vitro).	**Anti-inflammatory effect** All fractions suppressed lymphocyte proliferation (dichloromethane fraction was the most potent, with an IC_50_ of 19.8 µg/mL). The butanol fraction at 100 µg/mL reduced inflammatory cytokine levels, specifically IL-4 and IFN-γ.	[63]
**Fruits**	Essential oil	The washed platelets in rabbits (In vitro).	**Anticoagulant effect** (+)-Fenchone and estragole at 10 and 5 µg/mL showed significantly high inhibition of collagen-induced platelet aggregation, with (+)-fenchone exhibiting 93.5% and 58.4% inhibition, respectively, and estragole exhibiting 98.7% and 54.6% inhibition, respectively.	[64]
**Fruits and aerial parts**	Essential oil (10, 30, and 100 mg/kg/day); anethole (1, 3, 10, or 30 mg/kg/day)	Guinea pig plasma (In vitro). Acute pulmonary thromboembolism (In vivo).	**Anticoagulant and antithrombotic effect** Fennel oil and anethole showed the following effects: significant inhibition of arachidonic acid, collagen, ADP, and U46619-induced platelet aggregation (IC_50_ from 4 to 147 g/mL); prevention of thrombin-induced clot retraction; and protection against collagen-epinephrine-induced paralysis at 30 mg/kg, with 70% and 83% protection, respectively.	[41]
**Seeds**	Fennel powder (300 mg/kg b.w)	Obese male albino rats (In vivo).	**Hypolipidemic effect** Significant decrease in body weight. Significant decrease in albumin levels and total protein. Significant decrease in TC and TG. Significant increase in HDL-chol and decrease in LDL-chol. Significant decrease in ALT, AST, and ALP, MDA and MPO.	[65]
**Seeds**	Aqueous extract (50,100, and 200 mg/kg. i.p)	Male BALB/c mice fed a high cholesterol (In vivo).	**Hypolipidemic effect** Significant decrease in TC at 100 mg/kg; triglycerides at 100 and 200 mg/kg, and LDL at 50 and 100 mg/kg. However, HDL enhanced significantly at 100 mg/kg.	[11]
**Seeds**	Hydroalcoholic extract (150 mg/kg b.w for 3 weeks)	Male rats fed a high cholesterol regimen (In vivo).	**Hypolipidemic effect** Significant reduction in TG, TC, LDL, and elevation in HDL. Significant decrease in ALP and ALT levels.	[66]
**Seeds**	Methanolic extract (100 mg/kg/day)	Swiss albino mice exposed to 2-Gy gamma irradiation (In vivo). DPPH radical scavenging (In vitro).	**Antioxidant effect** Significant decrease in MDA, Significant increase in SOD and CAT levels. Fennel extract completely inhibited DPPH radicals, showing 100% scavenging activity at a concentration equivalent to 29.64 mg/g of total phenolic compounds in dry matter.	[67]
**Seeds**	Distilled water, ethanolic (80%), and acetonic (80%)	FTC, β-carotene, and ABTS assays (In vitro).	**Antioxidant effect** A significant antioxidant effect was observed with the distilled water extract (48.35 ± 0.19%), followed by the ethanol (45.10 ± 0.34%) and acetone (28.45 ± 0.11%) extracts in the FTC assay. Distilled water showed the higher protection against β-carotene bleaching (66.63 ± 0.05%), followed by ethanol (66.63 ± 0.05%) and acetone (58.11 ± 0.11%). Acetone extract exhibits the greatest ABTS value (7.28 ± 0.17 mM TE/g), followed by ethanol (5.70 ± 0.27 mM TE/g) and distilled water (4.26 ± 0.028 mM TE/g).	[68]
**Aerial parts**	Essential oil (1000, 750, 500, 250, and 100 ppm)	TBARS assay and micellar model system (In vitro).	**Antioxidant effect** Strong antioxidant activity that α-tocopherol at all concentrations. Inhibiting the peroxidation of linoleic acid. Reduction of the formation of hydroperoxydienes.	[69]
**Leaves**	Essential oil (1.5 to 24 mg/mL), diethyl ether (40 to 400 mg/mL), and ethyl acetate (28 to 160 mg/mL)	DPPH assay (In vitro).	**Antioxidant effect** Potential antioxidant activity compared to ascorbic acid was observed for the essential oil (IC_50_: 900 µg/mL), diethyl ether extract (IC_50_: 6.2 µg/mL), and ethyl acetate extract (IC_50_: 1.5 µg/mL).	[70]
**Leaves**	Essential oils (10 µg/mL)	DPPH assay (In vitro).	**Antioxidant effect** The weak DPPH scavenging ability of the samples may be attributed to their anethole content.	[71]
**Fruits**	Coumarins (30 µM)	DPPH and ABTS free radical scavenging activities (In vitro).	**Antioxidant effect** Scopoletin (48.34%), 8-methoxypsoralen (51.57%), bergapten (49.89%), and imperatorin (50.73%), significantly inhibited DPPH. Their corresponding ABTS radical scavenging activities were 47.05%, 50.53%, 50.44%, and 50.27%, respectively.	[37]
**Leaves, stems, shoots, and inflorescences**	Methanolic extract (0.15–20 mg/mL)	DPPH scavenging, reducing power, and inhibition of β-carotene bleaching assays (In vitro).	**Antioxidant effect** DPPH radical scavenging increases with the concentration increase in shoots, leaves, and inflorescences extracts (>50% at 10 mg/mL). Reducing power rose with concentration, reaching excellent levels for shoot, inflorescence, and leaf extracts at 5 mg/mL. Shoot and leaf extracts exhibited the most potent β-carotene bleaching inhibition (>90% at 20 mg/mL).	[72]
**Aerial parts and fruits**	Essential oils 1–24 g/L (DPPH) 100–2000 mg/L (TBARS and hydroxyl radical) 50–250 mg/L (lipoxygenase)	DPPH, TBARS, H_2_O_2_ radical scavenging activity, lipoxygenase assays (In vitro).	**Antioxidant effect** At the highest concentrations (12–24 g/L), the DPPH free radical scavenging capacity is >85%. At the lowest concentrations (100 and 250 mg/L), the fruit oils showed lower activity than the oils obtained from the aerial parts in the TRABS assay. Neither the oils from the aerial parts nor the fruit showed hydroxyl radical scavenging capacity > 50%. A stronger 5-lipoxygenase inhibition was observed for the essential oils tested at 250 mg/L.	[73]
**Leaves**	Essential oils 1–250 µg/mL (ABTS and H_2_O_2_) 1–200 µg/mL (Antioxidant Enzymes Activity)	ABTS and H_2_O_2_ radicals scavenging assays (In vitro). ROS generation and antioxidant enzymes activity on polymorphonuclear leukocytes (PMN) (In vitro).	**Antioxidant effect** EO induced 50% reduction in ABTS and H_2_O_2_ radicals with IC_50_ value > 100 µg/mL, respectively. A significant reduction of the ROS levels in PMN treated with 100 and 200 µg/mL of EO. Increase in CAT, SOD, and GPx with increasing EO concentrations.	[74]
**Seeds**	Hydro-methanolic extract (10–100 µg/mL)	H_2_O_2_ radical scavenging activity or Fenton reaction (In vitro).	**Antioxidant effect** H_2_O_2_ radical scavenging activity of the extract increased with concentration, reaching 82.64 ± 0.13% inhibition at 100 µg/mL.	[75]
**Fruits**	Essential oil (0.75–10 mg/mL). 100 and 200 mg/kg/day (In vivo)	TAC, DPPH, ABTS, FRAP assays (In vitro). The bleomycin (BLM)-induced pulmonary fibrosis assay (In vivo).	**Antioxidant effect** TAC assay showed good antioxidant potential with a value of 7.26 ± 0.34 mg GAE/g FEO. FRAP assay revealed significant reducing power with EC_50_ = 63.44 ± 2.29 mg/mL. DPPH radical scavenging activity is comparable to that of BHT. ABTS activity was lower than Trolox. Fennel oil has been shown to decrease MDA levels while enhancing the activities of SOD and CAT enzymes.	[76]
**Seeds**	Aqueous extract (AEFv) and butanolic fraction (BFFv) (0.01–1.5 mg/mL)	DPPH, FRAP, and β-carotene bleaching assays (In vitro).	**Antioxidant effect** DPPH scavenging activity increased with concentration, peaking at 400 μg/mL. BFFv exhibited stronger antioxidant activity than AEFv, with an IC_50_ of 30.6 ± 0.61 μg/mL. BFFv and AEFv showed greater inhibition activity in the bleaching of β-carotene, with IC_50_ values of 0.24 ± 0.051 and 0.3 ± 0.047 mg/mL, respectively. The iron chelation assay demonstrated the ability of the AEFv and BFFv to reduce ferric ions to ferrous ions. This effect was proportional to the concentration tested.	[12]

**Table 2:** The summary of mechanisms and major bioactive compounds involved in cardiovascular activities.

Mechanisms of Action	Bioactive Compounds
Reduction of mean arterial pressure and heart rate	Estragole, trans-anethole, phenolic compounds
Inhibition of angiotensin-converting enzyme (ACE) activity	Trans-anethole, gallic acid, caffeic acid, *p*-coumaric acid, quercetin
Downregulation of angiotensin II receptor (AT1R) and related gene expression	Kaempferol, quercetin, trans-anethole, naringin
Enhancement of diuresis and electrolyte excretion (Na^+^, K^+^, Cl^−^, Ca^2+^)	Fenchone, chlorogenic acid, caffeic acid, isoquercitrin, (–)-epicatechin
Activation of the NO/sGC/cGMP vasorelaxant pathway	Caffeic acid, ferulic acid, coumaric acid, quercetin, kaempferol, naringenin
Activation of muscarinic receptors and endothelium-dependent relaxation	Phenolic acids, naringin
Inhibition of store-operated and voltage-dependent Ca^2+^ entry	Trans-anethole, essential oil constituents
Opening of potassium channels leading to hyperpolarization	Quercetin, kaempferol, naringenin
Enhancement of endogenous antioxidant defense (↑ SOD, ↑ CAT, ↑ GPx, ↑ GST, ↓ MDA, ↓ MPO, ↓ ROS)	Trans-anethole, caffeic acid, syringic acid, quercetin, catechin, S-limonene, kaempferol, α-pinene, (–)-fenchone, chlorogenic acid
Reduction of oxidative stress, DNA damage, and mitochondrial dysfunction; downregulation of IL-1β, TNF-α, IL-6	Caffeic acid, quercetin, catechin, anethole, ferulic acid, chlorogenic acid
Inhibition of collagen, ADP, and AA-induced platelet aggregation (↓ TXB_2_, ↓ PGE_2_); prolongation of PT; inhibition of fibrin formation and factor Xa activity	Trans-anethole, estragole, (+)-fenchone, quercetin, catechin, *p*-coumaric acid, chlorogenic acid, syringic acid
Inhibition of iNOS and COX-2 expression; suppression of NF-κB and MAPK signaling	Trans-anethole, estragole, quercetin, ferulic acid, chlorogenic acid, *p*-coumaric acid
Regulation of lipid metabolism: ↓ LDL, ↓ VLDL oxidation, ↓ SREBP-2 and LDLR expression, ↓ HMG-CoA reductase activity; ↑ HDL formation and LDL clearance	Trans-anethole, ferulic acid, *p*-coumaric acid, quercetin, catechin, hesperidin, isorhamnetin, limonene
Free radical scavenging and metal ion chelation (DPPH, ABTS, hydroxyl, superoxide assays; lipid peroxidation inhibition)	Quercetin, kaempferol, rutin, epicatechin, *p*-coumaric acid, ferulic acid, chlorogenic acid, syringic acid, scopoletin, bergapten, imperatorin

## Data Availability

No new data were created or analyzed in this study. Data sharing is not applicable to this article.

## Abbreviations

ACE	Angiotensin-Converting Enzyme
AKT	Protein Kinase B
ALP	Alkaline Phosphatase
ALT	Alanine Aminotransferase
AST	Aspartate Aminotransferase
CAT	Catalase
COX1/2	Cyclooxygenase-1/2
CPK	Creatine Phosphokinase
CREB	cAMP Response Element-Binding Protein
cGMP	Cyclic Guanosine Monophosphate
CRP	C-Reactive Protein
ERK1/2	Extracellular Signal-Regulated Kinases 1 and 2
FV	*Foeniculum vulagre* Mill.
GC-MS	Gas Chromatography-Mass Spectrometry
GPx	Glutathione Peroxidase
GSK-3β	Glycogen Synthase Kinase-3 Beta
GST	Glutathione S-Transferase
HepG2	Human Liver Cancer Cell Line G2
HMG-CoA	3-Hydroxy-3-Methylglutaryl-Coenzyme A
HPLC-DAD	High-Performance Liquid Chromatography with Diode Array Detection
IFN-γ	Interferon-Gamma
IKB-α	Inhibitor of Nuclear Factor Kappa-B Alpha
IL-1β	Interleukin-1 Beta
IL-4	Interleukin-4
IncRNA MIAT	Long Non-Coding RNA Myocardial Infarction Associated Transcript
iNOS	Inducible Nitric Oxide Synthase
JNK1	c-Jun N-terminal Kinase 1
LDH	Lactate Dehydrogenase
L-NAME	N-G-Nitro-L-Arginine Methyl Ester
MAPK	Mitogen-Activated Protein Kinase
MPO	Myeloperoxidase
NF-κB	Nuclear Factor Kappa-Light-Chain-Enhancer of Activated B Cells
NLRP3	NOD-Like Receptor Pyrin Domain Containing 3
Nrf2	Nuclear Factor Erythroid 2-Related Factor 2
PMA	Phorbol 12-Myristate 13-Acetate
SOD	Superoxide Dismutase
SREBP-2	Sterol Regulatory Element-Binding Protein 2
TNF-α	Tumor Necrosis Factor Alpha
